# PAGD: the *Persea americana* Genome Database and a Docker-based transcriptome analysis workflow

**DOI:** 10.1186/s12864-026-12924-3

**Published:** 2026-06-01

**Authors:** Siyi Ma, Huadong Feng, Danni Yang, Zhenzhen Wu, Ruiyuan Li, Xin Geng, Chenyi Shi, Shimeng Liu, Yunqiang Yang, Chengjun Zhang

**Affiliations:** 1 Institute of International Rivers and Eco-security, Yunnan University, Kunming, Yunnan 650091 China; 2 Kunming Institute of Botany, Chinese Academy of Sciences, Kunming, Yunnan 650201 China; 3 National Key Laboratory for Development and Utilization of Forest Food Resources, Zhejiang A&F University, Hangzhou, 311300 China; 4 Xishuangbanna Tropical Botanical Garden, Chinese Academy of Sciences, Kunming, Yunnan 650223 China; 5 Jiaxing Synbiolab Biotechnology Co., Ltd, Jiaxing, 314006 China

**Keywords:** *Persea americana*, Database, Drupal, Tripal, Transcriptome, Docker, Snakemake

## Abstract

**Background:**

Avocado (*Persea americana*) is an economically important fruit with growing global production. While multiple genome assemblies and transcriptomic datasets are publicly available—including the dedicated platform AvoBase—these resources remain fragmented and lack integration with pre‑computed multi‑omics analyses and reproducible workflows.

**Results:**

We present the *Persea americana* Genome Database (PAGD; http://bioinfor.kib.ac.cn/), an integrated platform consolidating two high‑quality genome assemblies (Hass chromosome‑level and West Indian telomere‑to‑telomere). It also hosts RNA‑seq datasets from 13 NCBI BioProjects, each linked to detailed biosample metadata, all uniformly re‑processed. Pre‑computed results include gene family classification (68 TPS genes), collinearity, gene density, and expression profiles. PAGD offers BLAST, JBrowse, interactive heatmaps, and data download. Additionally, we developed three Docker‑encapsulated Snakemake workflows for reference‑based and reference‑free transcriptome analysis, eliminating manual software configuration.

**Conclusion:**

PAGD advances existing avocado genomic resources by integrating multi‑omics data with pre‑computed analyses and a reproducible transcriptome workflow. The encapsulated workflows lower the technical barrier for RNA‑seq analysis, are adaptable to other plant species, and support functional genomics, breeding, and comparative studies.

**Supplementary Information:**

The online version contains supplementary material available at 10.1186/s12864-026-12924-3.

## Background

*Persea americana*, commonly known as avocado or alligator pear, is an evergreen tree belonging to the Lauraceae family [1]. Native to Central America, avocado comprises three major ecological races: Mexican (*P*. *americana* var. *drymifolia*), Guatemalan (*P*. *americana* var. *guatemalensis*), and West Indian (*P*. *americana* var. *americana*) [2]. The ‘*Hass*’ cultivar, a hybrid of Guatemalan and Mexican populations [3, 4], now dominates global production, accounting for approximately 95% of the market due to its superior fruit quality and post-harvest characteristics [5, 6]. With global production increasing from 2.93 million tons in 2002 to 8.98 million tons in 2022—a threefold increase over two decades (FAO, 2023), avocado has become an economically significant crop. Moreover, due to its content of various vitamins, unsaturated fatty acids, and other beneficial nutrients, avocado has gradually gained popularity [7–10]. In terms of agricultural resource variety information, the UC Riverside variety collection (https://avocado.ucr.edu/), providing valuable references for both cultivation practices and scientific research.

The past decade has witnessed substantial progress in avocado genomics. Early molecular marker and deep sequencing of transcriptome studies laid the foundation for understanding avocado genetic diversity and domestication history [11–15]. The first draft genome of the ‘*Hass*’ cultivar, though fragmented, provided initial insights into avocado genome architecture [3, 16]. Subsequently, chromosome-level assemblies became available for both ‘*Hass*’ [17] and the ‘*Gwen*’ cultivar [18], with the latter providing evidence for three separate domestication events and identifying selective sweep targets related to fruit development [18]. Most recently, a telomere-to-telomere (T2T) assembly of the West Indian avocado reached 841.6 Mb with 12 gap-free chromosomes [19], while the Mexican race genome has also been sequenced [3]. Meanwhile, the abundance of genomic resources [20] has facilitated the establishment of an extensive SNP genotype database, which serves as a crucial tool for population genetics and genome-wide association studies [21]. Despite these advances, avocado genomic resources remain fragmented across general-purpose repositories such as NCBI and Phytozome, where data are unfriendly for researchers to download and analysis.

While general-purpose databases like NCBI and Phytozome provide invaluable archival functions, they present inherent limitations for species-focused research. First, omics data for a given species are scattered across thousands of independent entries, requiring researchers to manually collect, curate, and harmonize datasets—a time-consuming and error-prone process. Second, these repositories serve primarily as data archives rather than analytical platforms, offering limited capacity for integrated visualization or cross-dataset comparisons. Third, the reproducibility of bioinformatics analyses is often compromised by undocumented software versions and parameters. Although the recently launched AvoBase (https://www.avocado.uma.es/easy_gdb/index.php) provides genome browsing and data download, it lacks integrated multi-omics analyses and reproducible workflow encapsulation. Consequently, there remains a critical need for a comprehensive, integrated platform that not only consolidates avocado multi-omics data but also provides ready-to-use analytical tools, the results after unified pre-computation and reproducible workflows to accelerate biological discovery.

To address this gap, we present the *Persea americana* Genome Database (PAGD, http://bioinfor.kib.ac.cn/)—a multi-omics platform integrating two high-quality genome assemblies (Hass chromosome-level [17] and West Indian T2T [19]), 310 transcriptomic datasets, and pre-computed analyses including gene families, collinearity blocks, and expression profiles. Beyond data integration, PAGD features user-friendly interfaces for BLAST [22], JBrowse [23], and dynamic heatmap visualization. A key innovation is the implementation of Docker-encapsulated Snakemake workflows for transcriptome analysis, enabling researchers to perform reproducible analyses without local software installation or programming expertise. PAGD thus serves as both a centralized data repository and an analytical ecosystem, designed to empower the avocado research community and accelerate translational research from genomics to breeding applications.

## Results

### PAGD: a multi-omics database for avocado research

#### Database content and architecture

PAGD serves as a comprehensive platform integrating multi-omics data for avocado research (Table 1). The database currently houses two high-quality genome assemblies: the Hass chromosome-level assembly (912.9 Mb, Nath et al. 2022) and the West Indian T2T assembly (841.6 Mb with 12 gap-free chromosomes, Yang et al. 2024). Both assemblies have been annotated, yielding 40,721 and 42,442 genes. In addition, genome data for the ‘*Gwen*’ cultivar [18] and Mexican race [3] are included for comparative analyses. In addition, information on 63 avocado cultivars has been collected, encompassing cultivar-specific characteristics such as agronomic traits and geographical adaptation data. This comprehensive information can be accessed in detail via the “Cultivar” section on the website’s homepage. To support interspecies studies, we have also integrated genomes of model species including *Populus trichocarpa* [24]and *Arabidopsis thaliana* [25].

**Table 1:** Data statistics in PAGD

Species	*P*. americana var. americana (West Indian T2T)	*P*.americana cv.‘Hass’ (Hass chromosome-level)	Populus trichocarpa	Arabidopsis thaliana
Gene Exon CDS mRNA ncRNA Protein Chromosomes RNA-seq Biosamples BAM	40,721 181,365 48,829 48,829 5183 48,829 12 9 9 3	42,442 228,370 42,442 42,442 4643 42,442 12 299 301 /	34,621 332,124 308,640 52,085 / 29,665 19 / / /	38,312 313,952 286,067 48,399 / 27,562 5 / / /

**Fig. 1:**
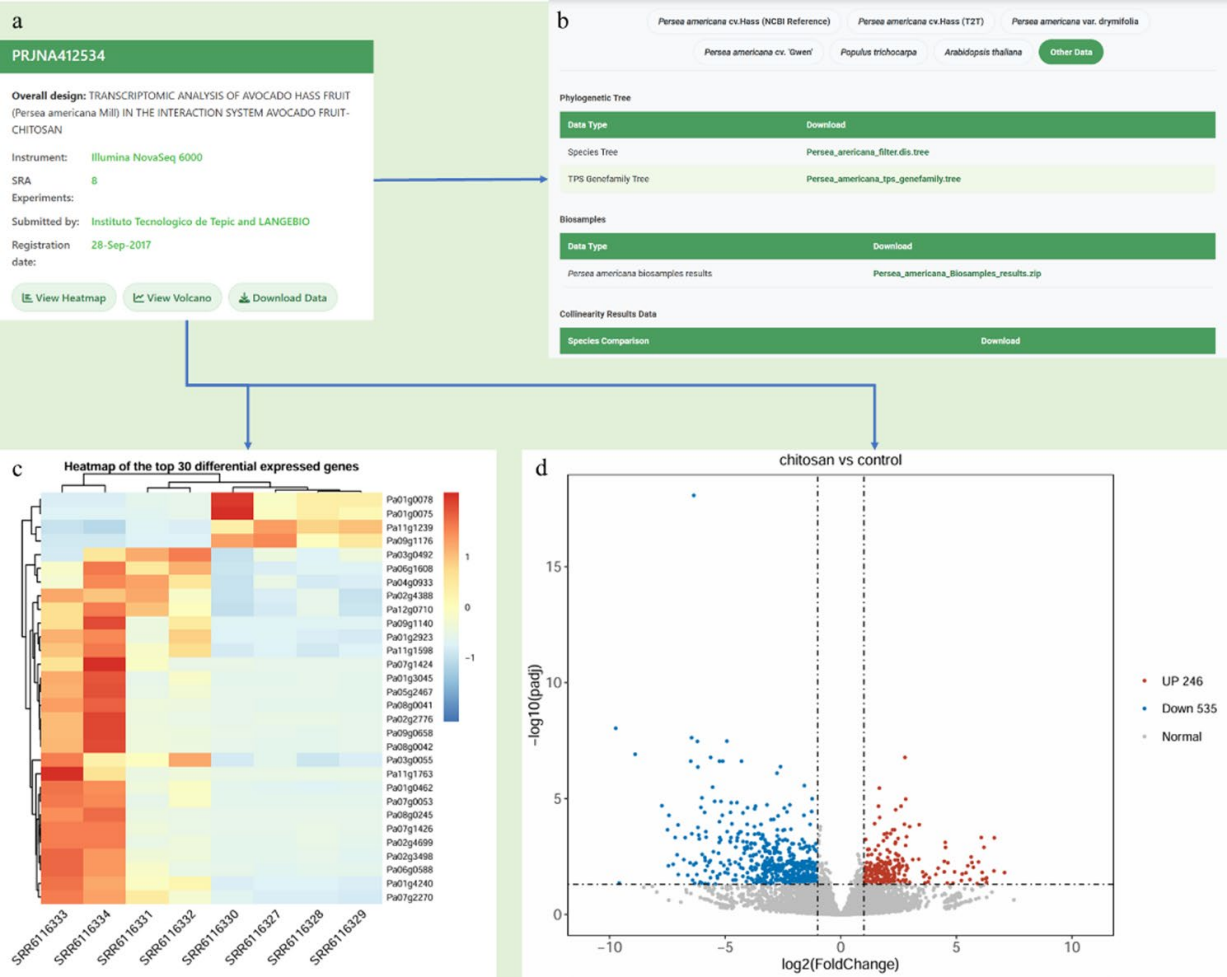
Analysis results and download of transcriptome expression levels and differentially expressed genes. (**a**) Transcriptome project information. (**b**) Data download page. (**c**) Heatmap of the top 30 differential expressed genes. (**d**) Volcano map of differentially expressed genes

For transcriptomic data, we curated 13 NCBI BioProjects, encompassing diverse biological conditions such as fruit development, chitosan treatment, Fusarium dieback stress, cold storage, and drought responses. All samples have been uniformly reprocessed using our standardized workflow (see below), generating expression matrices (FPKM, TPM, and count values) ready for cross-study comparisons (Fig. 1). Pre-computed analyses include gene family classifications (68 TPS genes), genome-wide collinearity blocks, gene density distributions, and KEGG/GO enrichment results, all accessible through the “Specific Analysis” section of the website.

**Fig. 2:**
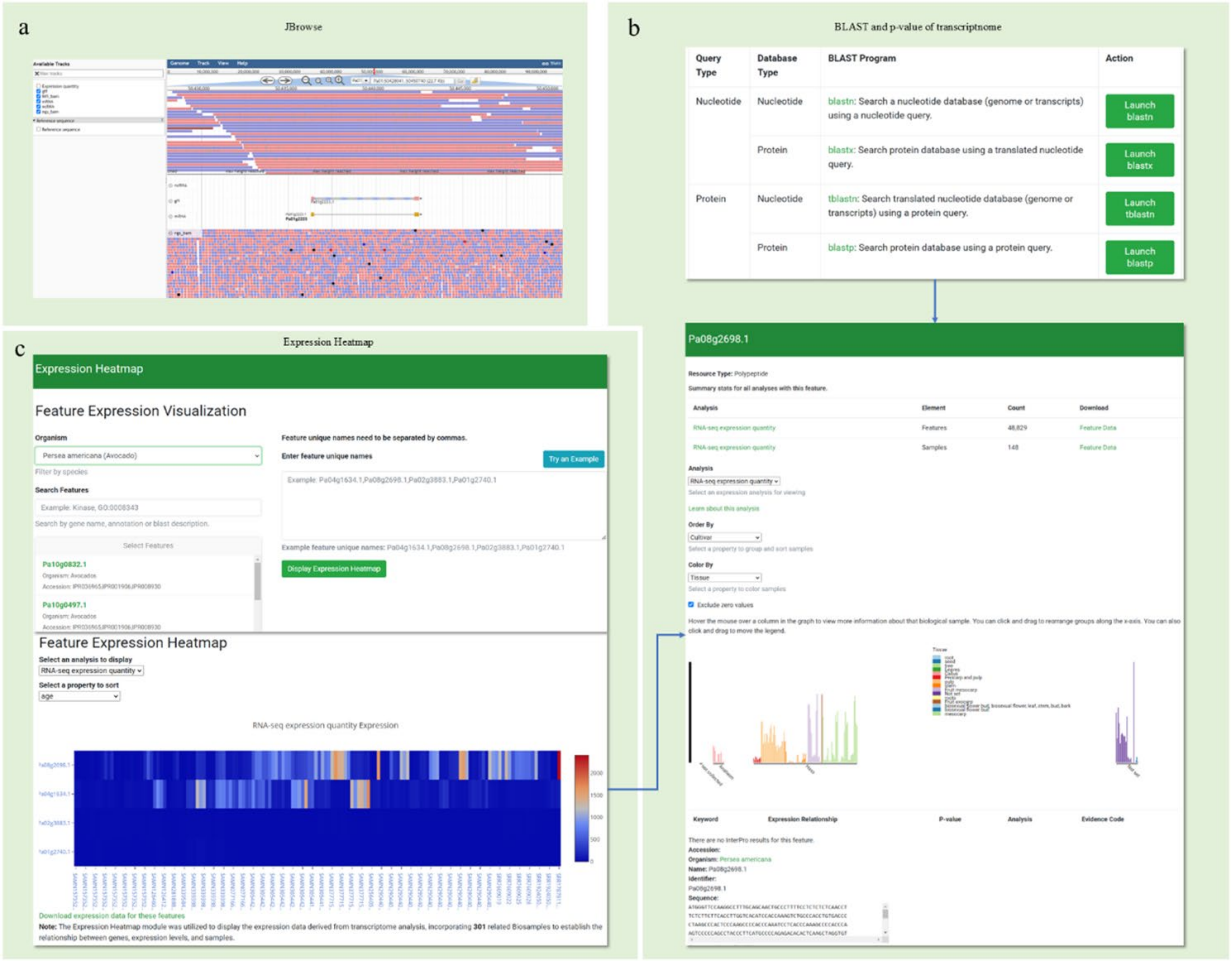
The main tools and data presentation functions of PAGD. (**a**) JBrowse. (**b**) BLAST and *P*-value of transcriptome. (**c**) Expression Heatmap

**Fig. 3:**
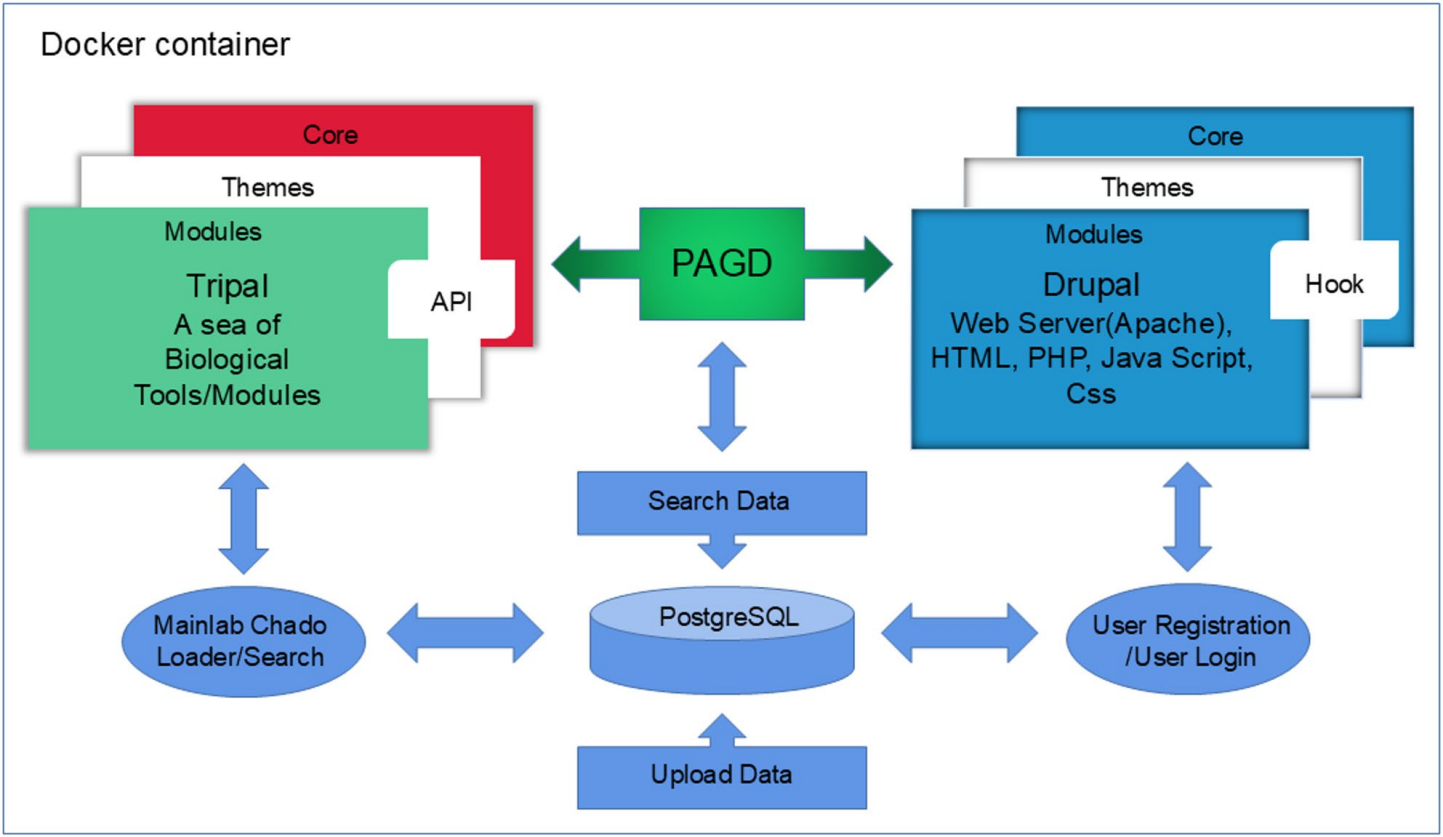
The infrastructure of PAGD. PAGD is built upon the Drupal framework. It leverages Tripal to interlink and visually present multi-omics data, including genomics. The underlying data and information are stored in a PostgreSQL database. Furthermore, the entire system is encapsulated within Docker containers to guarantee stability and enhanced security

The database architecture is built on Drupal (https://www.drupal.org/) and Tripal (https://tripal.info/), with PostgreSQL (https://www.postgresql.org/) as the backend. Key visualization tools include JBrowse for genome browsing (Fig. 2a), BLAST for sequence alignment (Fig. 2b), and dynamic heatmaps for expression data (Fig. 2c). A system architecture diagram illustrating the integration of data storage, analysis workflows, and user interface components is provided in Fig. 3.

#### Validation of core analytical tools: expression heatmap module

To assess the functionality and reliability of the expression heatmap module implemented in the avocado genome database, we performed validation tests focusing on its two primary features: single-gene expression visualization and user‑customized multi‑gene heatmap generation. The module, based on the Tripal Analysis Expression extension originally developed for the Harwood Genome Database (HGD) [26], stores and visualizes normalized gene expression values derived from RNA‑seq experiments.

Single‑gene expression visualization. For any gene or mRNA feature with associated expression data, an “Analysis” block appears in the middle of the feature page. Selecting “RNA‑seq expression quantity” displays an interactive bar chart or one‑dimensional heatmap generated using the D3.js and plotly.js libraries. We examined expression patterns for four avocado genes (*Pa10g0832.1*, *Pa10g0497.1*, *Pa07g1693.1*, *Pa06g1667.1*). In each case, the module correctly retrieved the corresponding expression values from the Chado database and rendered the graphical representation without error. Hovering over individual bars or heatmap cells displayed the precise expression value and associated biomaterial metadata. Clicking on a biomaterial name navigated to the detailed biomaterial information page, which includes links to the corresponding NCBI BioSample and SRA experiment pages. Clicking on the transcript name within the heatmap view returned the user to the gene’s main page, where both the expression graph and InterPro functional annotations (where available) are displayed.

Multi‑gene heatmap generation. The module provides a web form that accepts a user‑supplied list of gene identifiers and generates a two‑dimensional heatmap showing expression levels across all available biomaterials. We tested this feature with gene lists of varying sizes (2 to 12 genes) from the avocado terpene synthase (TPS) gene family. For each list, the module successfully produced an interactive heatmap with correctly normalized expression values. All interactive features—zoom, pan, hover information display, and clickable elements—functioned as expected. Clicking on a gene name in the heatmap redirected to the corresponding gene page, where the single‑gene expression visualization could be examined; clicking on a biomaterial name redirected to the biomaterial details page, confirming the integrity of cross‑linking between data types.

Data provenance and external links. The biomaterial detail pages provide direct hyperlinks to the original NCBI BioSample and SRA records, enabling users to trace the experimental conditions and raw data underlying each expression measurement. This ensures that all expression data presented in the heatmaps are accompanied by complete provenance information.

Together, these tests demonstrate that the expression heatmap module reliably visualizes gene expression data, supports user‑defined queries, and maintains accurate cross‑references to both internal feature pages and external public repositories. The module therefore provides a functional tool for exploring avocado transcriptome data.

#### Biological insights derived from PAGD: TPS gene family analysis

To demonstrate the database’s utility for biological discovery, we conducted an in-depth analysis of the terpene synthase (TPS) gene family—a key group of enzymes involved in plant defense, signaling, and pollinator attraction [27–29].

Identification and phylogenetic classification: Using HMM profiles of conserved TPS domains, we identified 68 TPS genes (representing 93 transcripts including splice variants) in the West Indian T2T genome assembly. Phylogenetic analysis was performed by aligning the full-length protein sequences with TPS genes from *A*. *thaliana* [30], followed by maximum likelihood tree construction (Figure S1). Based on phylogenetic relationships and conserved domain architecture, the majority of avocado TPS genes were classified into two major subfamilies: TPS‑a and TPS‑b. The few genes could not be confidently assigned to any known angiosperm TPS subfamily (TPS‑a, ‑b, ‑c, ‑e/f, ‑g, or ‑h) and are thus provisionally categorized as “Others”. These may represent lineage‑specific expansions or divergent members that are yet to be functionally characterized. Furthermore, no members belonging to the TPS‑c, TPS‑d, TPS‑e/f, or TPS‑h subfamilies were identified.

Chromosomal distribution and gene expansion: Mapping the 68 TPS genes onto the 12 avocado chromosomes revealed a non‑random distribution (http://bioinfor.kib.ac.cn/tps_genefamily). Chromosome 10 harbored the highest density of TPS genes (27 genes), followed by chromosome 6 (15 genes) and chromosome 3 (14 genes). Notably, multiple gene clusters were identified. On chromosome 10, two major clusters were observed: a proximal cluster comprising 7 genes and a distal cluster containing 20 genes. The distal cluster is predominantly composed of TPS‑b subfamily members. On chromosome 3, two separate clusters were detected, consisting of 8 and 6 genes, respectively; the 6‑gene cluster contains TPS‑a genes together with a few unclassified genes. Chromosome 6 contained a large cluster of 11 genes along with a smaller adjacent pair (2 genes); the 11‑gene cluster harbors TPS‑a genes as well as other TPS members. Additional smaller clusters were found on chromosome 5 (4 genes) and chromosome 7 (2 genes), which include TPS‑b genes. The remaining chromosomes (1, 2, 4, 9, and 12) each carried only one or two isolated genes.

Analysis of gene duplication mechanisms revealed that tandem duplication accounts for the majority of TPS gene expansion: 60 of the 68 genes (88.2%) are located in tandem arrays. An additional 5 genes (7.4%) show evidence of segmental duplication, while the remaining 3 genes (4.4%) are considered singleton duplicates. The predominance of tandem duplication suggests lineage‑specific expansion of the TPS gene family in avocado, with most members organized into multi‑gene clusters on chromosomes 3, 5, 6, 7, and 10.

Evolutionary implications: TPS‑b genes are primarily responsible for synthesizing monoterpenes [31]. The prominent TPS‑b cluster on chromosome 10, the largest gene cluster in the genome, likely arose through repeated tandem duplications and may reflect evolutionary adaptations to the species’ native environment. In this region, intense biotic pressures from herbivores and pathogens have probably driven the diversification of chemical defense mechanisms, with monoterpene synthases playing a central role. Meanwhile, the TPS‑a subfamily, involved in diterpene synthesis [31], exists exclusively on chromosomes 3 and 6. The presence of unclassified genes suggests that additional, perhaps avocado‑specific, terpene synthase functions await discovery.

These findings provide a foundation for future functional studies aimed at improving avocado fruit quality and disease resistance. All TPS‑related data—including gene id list, phylogenetic tree, chromosomal locations and KEGG/GO enrichment analysis results are freely accessible through the PAGD website (http://bioinfor.kib.ac.cn/tps_genefamily), enabling the research community to further explore this important gene family.

### Encapsulation of transcriptome workflow based on docker

#### Workflow architecture

To address the challenge of reproducible transcriptome analysis, particularly for researchers with limited bioinformatics expertise, we developed three standardized, Docker-containerized Snakemake workflows. These workflows are designed to accommodate both reference-based and reference-free analytical scenarios.

1. The HISAT2-based workflow sequentially performs quality control (FastQC/Fastp), read alignment (HISAT2), transcript assembly and quantification (StringTie), and concludes with differential expression analysis (DESeq2).
2. The STAR-based workflow conducts quality control, followed by alignment using STAR, gene-level quantification via HTSeq-count, and differential expression analysis with edgeR.
3. The reference-free workflow encompasses read alignment (Bowtie2), de novo transcriptome assembly (Trinity), expression quantification (RSEM), differential expression analysis (DESeq2).

#### Quantitative validation and benchmarking

To rigorously evaluate workflow performance and reproducibility, we conducted comprehensive benchmarking using 4 RNA-seq samples from avocado BioProject PRJNA849223. Three independent replicate runs of a representative dataset (four samples, ~ 124 M paired-end reads total) were performed to assess run-to-run variability.

Alignment efficiency: across 4 test samples, the HISAT2-based workflow achieved an average overall alignment rate of 93.6% (range: 92.4–94.5%), while the STAR-based workflow showed slightly higher rates (average 94.1%, range 93.3–94.8%). These rates are higher than published avocado transcriptome studies [32].

Reproducibility: Three independent runs on identical input data demonstrated exceptional reproducibility. For the HISAT2 workflow, gene-level TPM matrices showed near-perfect correlation between runs (Pearson’s *R* = 0.995 ± 0.001). Peak memory usage was highly consistent at 42.08 ± 0.01 GB (CV = 0.02%), and runtime varied by only 21.9 ± 0.6 min (CV = 2.9%). The STAR workflow exhibited similar stability: 43.2 ± 0.6 min runtime (CV = 1.5%) and peak memory of 20.9 ± 0.4 GB (CV = 1.8%) (Table S1).

Runtime and resource utilization: Complete analysis of four representative samples required 21.9 min for the HISAT2 workflow with peak memory of 42.1 GB. The STAR workflow required 43.2 min but used only 20.9 GB peak memory—a 50% reduction. Total CPU time averaged 5.5 h for HISAT2 and 6.8 h for STAR, with CPU utilization of 1704% and 970%, respectively (Table S1).

To further evaluate the ability of the workflow to produce biologically meaningful results, we re‑analyzed a published RNA‑seq dataset investigating the effects of 1‑methylcyclopropene (1‑MCP) treatment on postharvest avocado fruit during cold storage (PRJNA849223). The original study examined Hass avocado fruit with high dry matter (HDM; ~27%) treated with 1‑MCP at different time points (0, 14, and 21 days) and stored at 5 °C. We focused on two comparisons: 0 d vs. 14 d and 0 d vs. 21 d, each with three biological replicates per condition (accessions: SRR19654120‑22, SRR19654116‑18 for 0 d vs. 14 d; SRR19654120‑22, SRR19654113‑15 for 0 d vs. 21 d). Using the same HISAT2‑based quantification and DESeq2 pipeline described above, we identified differentially expressed genes (DEGs; |log2FC| > 1, FDR < 0.05). In the 0 d vs. 14 d comparison, 3985 DEGs were detected (2081 upregulated, 1904 downregulated) (Table S2). For 0 d vs. 21 d, the number increased to 12,494 DEGs (5820 upregulated, 6674 downregulated) (Table S3). Although the absolute numbers differ from those reported in the original study (1121 DEGs for 0 d vs. 14 d and 5279 for 0 d vs. 21 d), likely due to differences in analysis parameters or genome annotation versions, the overall trend is consistent: prolonged cold storage leads to a substantially larger transcriptional response [32]. GO and KEGG enrichment analyses revealed that the differentially expressed genes were significantly enriched in ribosome-related pathways (e.g., structural constituent of ribosome, ribosome biogenesis, and KEGG 03010 Ribosome), indicating translational reprogramming under cold stress, as well as in the negative regulation of ethylene-activated signaling pathway, consistent with the well‑documented role of 1‑MCP in inhibiting ethylene responses during postharvest storage [32]. These results confirm that the workflow can reproduce key biological conclusions from independent studies, supporting their utility for hypothesis generation in avocado research.

#### Cross-species applicability

To evaluate workflow versatility beyond avocado, we applied the reference-based workflows to publicly available RNA-seq data from three additional plant genera: *Rhododendron* (Ericaceae), *Cinnamomum* (Lauraceae), and *Torreya* (Taxaceae). These species span a wide range of genome sizes (from ~ 500 Mb in *Rhododendron* to > 10 Gb in *Torreya*) and evolutionary distances.

Across all tested datasets, the workflows executed without errors. In HISAT2-based alignment tests across three genera (Rhododendron, Ericaceae; Cinnamomum, Lauraceae; Torreya, Taxaceae), the workflow achieved over 90% overall alignment rates for all datasets. For Torreya, despite its large genome size (~ 19 Gb), the pipeline efficiently processed the data and gene expression levels and DEGs.

#### Availability and usability

All three transcriptome analysis workflows (HISAT2-based, STAR-based, and reference-free Trinity-based) are publicly available as Docker images on Docker Hub (https://hub.docker.com/r/siyinobelma/, siyinobelma/hisat-snake:1.2, siyinobelma/star-snake:1.1, siyinobelma/denovo-snake:1.2). The complete repository on GitHub (https://github.com/siyinobelma/transcript-analysis-workflow) hosts a README document, configuration files, sample data, and result demonstrations. Users can execute the full analysis workflow with a single command after pulling the image, eliminating the need for manual software installation. The standardized output directory structure (QC/, fastp/, mapping/, quantity/, DEA/) ensures that results are easily navigable, even for researchers with limited computational background.

## Discussion

PAGD represents an integrated multi-omics platform for avocado research, consolidating two high-quality genome assemblies, 310 uniformly re-processed transcriptome samples from 13 BioProjects, and pre-computed analytical results. By addressing the fragmentation of avocado genomic data across general-purpose repositories, PAGD provides a centralized resource that complements existing platforms such as AvoBase. While AvoBase offers genome browsing functionality, PAGD extends beyond visualization by integrating multi-omics data with pre-computed analyses and reproducible workflows.

The TPS gene family analysis demonstrates the utility of PAGD for biological discovery. Identification of 68 TPS genes in the West Indian T2T assembly, with 88.2% arising from tandem duplications, reveals lineage-specific expansion patterns. The prominent TPS‑b cluster on chromosome 10—the largest gene cluster in the genome—suggests adaptive evolution potentially related to monoterpene-mediated defense mechanisms. These findings, accessible through the PAGD website, provide a foundation for functional studies aimed at improving disease resistance and fruit quality traits in avocado breeding programs.

The Docker-encapsulated Snakemake workflows address a common challenge in transcriptome analysis: software configuration and reproducibility. Quantitative validation demonstrated high alignment rates (93.6–94.1%) and stable resource utilization across independent replicates. Analysis of cold stress data recovered known cold-responsive markers and revealed enrichment in ribosome biogenesis and ethylene signaling pathways, confirming biological relevance. Cross-species testing across Rhododendron, Cinnamomum, and Torreya—spanning genome sizes from ~ 500 Mb to > 10 Gb—indicates broad applicability beyond avocado.

Several limitations should be acknowledged. First, while transcriptome data have been uniformly re-processed, experimental heterogeneity across original studies may still influence cross-dataset comparisons. Second, the current version focuses primarily on genomic and transcriptomic data; future integration of proteomic, metabolomic, and phenotypic data would further enhance the platform’s value. Third, the integrated biological analysis tools of the database are still a bit lacking.

Despite these limitations, PAGD offers a practical resource for the avocado research community. By providing pre-computed analyses alongside raw data, it reduces redundant computational efforts and enables researchers to focus on biological interpretation. The encapsulated workflows lower technical barriers for laboratories with limited bioinformatics capacity, facilitating broader participation in avocado genomics research.

## Conclusions

PAGD is an integrated multi-omics platform that consolidates two high-quality genome assemblies, uniformly re-processed transcriptome data from 13 BioProjects, and pre-computed analyses including gene families, collinearity, and expression profiles. The database provides user-friendly tools for genome browsing, sequence alignment, and expression visualization, alongside data download options.

The TPS gene family analysis illustrates the platform’s utility for biological discovery, revealing lineage-specific expansion patterns and genomic organization that inform future functional studies. The Docker-encapsulated Snakemake workflows enable reproducible transcriptome analysis without manual software configuration, with validation demonstrating high alignment rates, reproducibility, and cross-species applicability.

PAGD thus serves as a centralized resource supporting avocado functional genomics, breeding research, and comparative studies. Future updates will incorporate additional data types—including SNP, QTL, and phenotypic information. We will conduct a comprehensive backup of containers and images every three months to ensure their continuous operation. We welcome community feedback to guide continued development and maintain PAGD as a sustainable resource for avocado research.

## Methods

### PAGD construction and implementation

PAGD was architected using a three-tiered framework prioritizing data integrity, service accessibility, and deployment scalability. At the foundational Data Tier, PostgreSQL 9.12 (https://www.postgresql.org/) implemented secure storage of multimodal genomic datasets—including nucleotide sequences, functional annotations, and experimental metadata—enforcing strict role-based access controls and ACID-compliant transactions. The Application Tier integrated Drupal 7.64 (https://new.drupal.org/home) with Tripal 3.10 (https://tripal.info/) to orchestrate biological data modeling and user interactions; this layer dynamically managed HTTP requests through customizable modules (Views for data presentation, CTools for UI components, Pathauto for semantic URL generation) while maintaining RESTful API compatibility. The Container Tier, deployed via Docker Compose on a CentOS 7.6 kernel, encapsulated runtime dependencies including Apache 2.4.6 (https://apache.org/) webserver and PHP 7.1.33 (https://www.php.net/) runtime, ensuring environment consistency across development and production stages.

System provisioning leveraged Docker containerization to orchestrate interdependent services. Core functionality required precise dependency management through Composer 1.8.4, validating compatibility across the LAMP stack. Biological data abstraction was achieved through Tripal’s Chado schema integration (tripal_chado), establishing relational mappings between genomic entities (genes, transcripts), taxonomic hierarchies (tripal_organism), and analytical pipelines (tripal_analysis). Specialized modules enabled domain-specific functionalities: tripal_feature curated sequence feature annotations, tripal_blast integrated BLAST homology search, and tripal_jbrowse embedded interactive genome browsers. Configuration workflows established granular user permissions while PHP extensions were optimized to fulfill Tripal’s computational requirements, with all schema constraints validated during Chado database initialization.

The comprehensive site search function (Mainlab Chado Search) includes “Gene Search”, “mRNA Search”, “Biological Sample Search” and “Protein Search” [33].

The download feature offers access to genome and annotation data in FASTA/FNA and GFF3/GBFF formats, as well as pre-computed analysis results including collinearity, gene density, and evolutionary trees.

### TPS gene family identification and analysis

protein sequences from 12 representative plant species were retrieved from the Phytozome database (v14, https://phytozome-next.jgi.doe.gov/). The selected species included: *A*. *thaliana*, *Oryza sativa*, *Zea mays*, *Populus trichocarpa*, *Theobroma cacao*, *Selaginella moellendorffii*, *Physcomitrium patens*, *Marchantia polymorpha*, *Chlamydomonas reinhardtii*, *Volvox carteri*, *Gonium pectorale*, and *Micromonas commoda*.

Based on previously established conserved domains, the TPS gene family was identified using Hidden Markov Model (HMM) profiles of the PF01397 (Terpene synthase family, metal-binding domain) and PF03936 (Terpene synthase family, C-terminal domain) motifs [34–36]. The seed files for these domains were downloaded from the Pfam database (http://pfam.xfam.org/). HMMER 3.0 (http://hmmer.org/) was employed to search against the protein dataset of the *P*. *americana* West Indian T2T genome assembly using the built HMM profiles with an E-value threshold of 1e-5. All candidate sequences containing either PF01397 or PF03936 were initially retained. These candidates were subsequently validated by searching against the Pfam and SMART databases(https://smart.embl-heidelberg.de/) to confirm the presence of both conserved domains. After manual inspection, sequences lacking a complete open reading frame (ORF) or representing obvious false positives were removed, yielding a final set of high-confidence TPS genes.

To classify the identified avocado TPS genes into known subfamilies (TPS-a, TPS-b, TPS-c, TPS-e/f, TPS-g, and TPS-h), a phylogenetic tree was constructed integrating TPS protein sequences from avocado and the 12 reference species, with a particular focus on previously characterized TPS sequences from *A*. *thaliana* [28]. Reference Arabidopsis TPS protein sequences were obtained from The Arabidopsis Information Resource (TAIR, https://www.arabidopsis.org/).

Multiple sequence alignment of all TPS protein sequences was performed using MAFFT (v7.490) [37] with the --auto option. The resulting alignment was then used to construct a maximum likelihood (ML) phylogenetic tree with IQ-TREE (v2.1.4-beta) [38]. The best-fit substitution model was automatically selected by the software using the -m TEST option. Branch support was assessed using the ultrafast bootstrap approximation with 1000 replicates (-bb 1000). The resulting tree file (in Newick format) was visualized and annotated using the iTOL (https://itol.embl.de/).

Subfamily classification of avocado TPS genes was performed based on their phylogenetic clustering with reference TPS sequences of known subfamilies from *A*. *thaliana*. The chromosomal locations of the identified avocado TPS genes were extracted from the genome annotation of the West Indian T2T assembly. A chromosomal distribution map was generated using the TBtools [39] to visualize the physical positions of TPS genes across the 12 avocado chromosomes.

### Collinearity analysis

Genome-wide collinearity analysis was performed to investigate syntenic relationships within the *P*. *americana* genome and between *P*. *americana* and representative model species. The analysis was conducted using MCScanX [40] (https://github.com/wyp1125/MCScanx). BLASTP search was first performed using the protein sequences of each species against themselves and against the other species to identify homologous gene pairs. The BLASTP output (in tabular format, -outfmt 6) was then used as input for MCScanX together with the GFF3 files. MCScanX was run with default parameters to identify collinear blocks based on the distribution of homologous genes along chromosomes.

The collinearity results were visualized using the SynVisio (https://synvisio.github.io/) [41]. For visualization, the following files were uploaded to the SynVisio platform: the MCScanX-generated .collinearity result file and the corresponding GFF3 annotation files for the species being compared.

### Chromosomal gene density calculation and visualization

To investigate the genome-wide distribution of genes across avocado chromosomes, gene density was calculated and visualized using the R package RIdeogram (https://github.com/TickingClock1992/RIdeogram) [42]. A karyotype file containing chromosome IDs and lengths was generated from the West Indian T2T genome assembly. Gene density was computed from the corresponding GFF3 annotation file using a sliding window approach with a window size of 1 Mb, and the resulting values were mapped onto the 12 avocado chromosomes. The ideogram was generated using a blue-yellow-red gradient to represent low, medium, and high gene density regions, respectively.

### GO and KEGG enrichment analyses of the TPS gene family

To investigate the potential biological functions and involved pathways of the identified TPS gene family in avocado, we performed Gene Ontology (GO) and Kyoto Encyclopedia of Genes and Genomes (KEGG) enrichment analyses. All analyses were based on the protein sequences of the avocado TPS genes. The complete protein sequences of all *P*. *americana* genes were subjected to functional annotation using the online version of eggNOG-mapper (http://eggnog-mapper.embl.de/) [43]. The analysis was configured with a taxonomic scope restricted to Viridiplantae (taxid: 33090) to ensure annotations were mapped to plant-specific pathways and terms. The resulting annotation file (out.emapper.annotations) was downloaded for subsequent processing. The raw annotation file from eggNOG-mapper was processed to generate standardized background annotation files suitable for enrichment analysis. This was accomplished using the eggNOG-mapper Helper tool, a utility within the TBtools (V2.111) [39].

GO enrichment analysis was performed using the GO Enrichment tool in TBtools [39]. The analysis was run with default parameters. The significance of enrichment for each GO term was calculated, and P-values were adjusted for multiple testing using the Benjamini-Hochberg (BH) method [44]. The output file contained detailed results, including the enriched GO terms, their associated P-values, and corrected P-values. Similarly, KEGG pathway enrichment analysis was conducted using the KEGG Enrichment Analysis tool within TBtools [39]. For both GO and KEGG enrichment results, significant terms were selected based on a threshold of adjusted P-value < 0.05. Bar plots were generated using the Enrichment Bar Plot tool in TBtools to visualize the most significantly enriched categories [39].

### Transcriptome workflow implementation and validation

All three workflows were independently encapsulated in separate Docker containers, each built upon the Ubuntu 20.04 base image. This design ensures consistent software dependency management and reproducible execution across diverse computing environments. Orchestration of all workflows is uniformly handled by Snakemake.

1. Docker-Encapsulated Transcriptome Analysis Workflow (HISAT2-based).

The container integrates a comprehensive suite of tools covering all stages of RNA-seq data processing. Raw data preprocessing is performed using FastQC (v0.11.9) [45] for initial quality assessment, followed by Fastp (v0.23.2) [46] for adapter trimming, quality filtering, and generation of quality control reports. For read alignment, HISAT2 (v2.2.1) [47] is employed to map clean reads to the reference genome, utilizing its hierarchical graph FM index (GFM) for efficient and accurate alignment of spliced transcripts. The resulting alignment files are processed and sorted using SAMtools (v1.16) [48].

Transcript assembly and quantification are conducted with StringTie (v2.2.1) [49], which assembles aligned reads into transcripts, estimates their abundances, and calculates expression levels in terms of fragments per kilobase of transcript per million mapped reads (FPKM) and transcripts per million (TPM). For comprehensive transcriptome exploration, the workflow also includes Cufflinks (v2.2.1) [50] and its associated utility gffread for alternative transcript assembly and format conversion.

The container further supports data retrieval and downstream analysis by integrating SRA Toolkit (v3.0.0) for direct download of public sequencing data from the NCBI Sequence Read Archive. For differential expression analysis, the R programming environment (v4.2.1) [51] is pre-configured with essential packages, including dplyr for data manipulation, ggplot2 for visualization, pheatmap for heatmap generation, and yaml for configuration file parsing. The Bioconductor package DESeq2 [8] is installed to perform statistical analysis of count-based expression data, identifying differentially expressed genes based on negative binomial distribution models.

2. STAR-HTSeq-edgeR Based Workflow.

The container integrates a complementary set of tools optimized for accurate splice-aware alignment and rigorous statistical analysis. Quality control and preprocessing are performed using FastQC (v0.11.9) [45] for initial assessment and Fastp (v0.23.2) [46] for adapter trimming and quality filtering. For read alignment, STAR (v2.7.10b) [52] is employed, utilizing its splice junction discovery algorithm and sequential maximum mappable seed search to achieve high alignment accuracy and speed, particularly suitable for mammalian-sized genomes. The generated alignment files are processed using SAMtools (v1.16) [48] for sorting and indexing.

Gene-level quantification is conducted with HTSeq-count (installed via pip) [53], which uses the aligned reads to count overlaps with genomic features based on the provided gene annotation file (GTF/GFF3 format), producing count matrices suitable for count-based statistical models. For differential expression analysis, the R environment (v4.2.1) [51] is pre-configured with edgeR [54], a Bioconductor package that employs empirical Bayes estimation and exact tests based on the negative binomial distribution to identify differentially expressed genes. Additional R packages, including dplyr for data manipulation, ggplot2 for visualization, and pheatmap for heatmap generation, are installed to support downstream analysis and result visualization. The workflow also includes Cufflinks (v2.2.1) [50] and its utility gffread for alternative transcript assembly when needed, as well as SRA Toolkit (v3.0.0) for direct data retrieval from public repositories.

3. Reference-Free (De Novo) Transcriptome Analysis Workflow.

The workflow begins with quality control and preprocessing using FastQC (v0.11.9) [45] and Fastp (v0.23.2) [46]. For de novo transcriptome assembly, Trinity (v2.15.1) [55] is employed, which reconstructs transcripts by sequentially assembling reads into contigs and then clustering related contigs based on shared read support. Prior to assembly, Bowtie2 (v2.4.4) [56] is used for initial read alignment to assess library quality, and Jellyfish (v2.3.0) [57] is utilized for k-mer counting to estimate optimal assembly parameters.

Following assembly, expression quantification is performed using RSEM (v1.3.3) [58] with Bowtie2, which maps reads back to the assembled transcriptome and estimates transcript abundances. For studies requiring rapid quantification, the container also includes Salmon (v1.9.0) [59] as an alternative alignment-free quantification method. Differential expression analysis is conducted using either DESeq2 [60] or edgeR [54] within the R environment (v4.2.1) [51], with additional R packages installed including dplyr, qvalue, fastcluster, and seqLogo to support comprehensive statistical analysis and visualization.

The workflow also incorporates ancillary tools essential for comprehensive transcriptome analysis: SAMtools (v1.16) [48] for manipulating alignment files, SQLite (v3.41.0), and MultiQC for aggregating quality control reports across multiple samples.

## Supplementary Information

Supplementary Material 1.

Supplementary Material 2.

Supplementary Material 3.

Supplementary Material 4.

## Data Availability

PAGD is freely available at http://bioinfor.kib.ac.cn/.

## Abbreviations

PAGD: the Persea americana Genome Database
BLAST: Basic Local Alignment Search Tool
TPS: Terpene Synthases
ORF: Open Reading Frame
KEGG: Kyoto Encyclopedia of Genes and Genomes
GO: Gene Ontology
SNP: Single Nucleotide Polymorphisms
