# Supplementary material for: PAGD: the Persea americana Genome Database and a Docker-based transcriptome analysis workflow
Source: BMC Genomics. 2026 Jun 1;27:652. doi: 10.1186/s12864-026-12924-3 (PMC13435527; doi:10.1186/s12864-026-12924-3)
Supplement: Supplementary file 1 [file 12864_2026_12924_MOESM4_ESM.docx]

Figure S1. The situation of the subfamilies of the TPS gene family: TPS-a is labeled in blue, and TPS-b is labeled in green.
